# Girl-Powered Nutrition Program: Key Themes from a Formative Evaluation of a Nutrition Program Co-designed and Implemented by Adolescent Girls in Low- and Middle-Income Countries

**DOI:** 10.1093/cdn/nzab083

**Published:** 2021-06-17

**Authors:** Elizabeth Dyke, Sophie Pénicaud, Jennifer Hatchard, Anne-Marie Dawson, Oresto Munishi, Chowdhury Jalal

**Affiliations:** Universalia Management Group, Westmount, Montreal, Quebec, Canada; Universalia Management Group, Westmount, Montreal, Quebec, Canada; Nutrition International, Ottawa, Ontario, Canada; Universalia Management Group, Westmount, Montreal, Quebec, Canada; Kilimanjaro Clinical Research Institute, Moshi, Tanzania; Nutrition International, Ottawa, Ontario, Canada

**Keywords:** implementation science/research, adolescents, girls, nutrition, evaluation, qualitative methods, gender equality, global health, program co-creation

## Abstract

**Background:**

To improve nutritional knowledge and attitudes of girls and young women, Nutrition International (NI) partnered with the World Association of Girl Guides and Girl Scouts (WAGGGS) to pilot the Girl-Powered Nutrition (GPN) program from 2018 to 2020 in 4 countries (Madagascar, the Philippines, Sri Lanka, and Tanzania).

**Objective:**

The aim was to share adolescent girls’ and programmers’ experiences with co-designing and implementing the GPN program in low- and middle-income countries (LMICs).

**Methods:**

A formative evaluation of the GPN program was commissioned by NI and undertaken by Universalia Management Group (Universalia). The evaluation was largely qualitative (employing focus groups, interviews, and document analysis). Based on the results of the formative evaluation, themes related to working with adolescent girls were identified.

**Results:**

The involvement of adolescents in the design, implementation, and evaluation of nutrition programming that targets them is essential for meaningful uptake. Sufficient time and respect must be given to the co-design process, including clearly defining adolescents’ roles, ensuring transparency and clear communication, and managing adolescents’ expectations. Ensuring adequate exposure and suitable timing for adolescent nutrition programming from adequately trained staff were identified as good practices from the evaluation. Program curriculum and activities must be appropriately tailored to adolescent age and stage, target adolescents and their gatekeepers and duty-bearers, and address the underlying issues of poverty, gender inequality, and structural norms that negatively impact adolescents’ agency and nutrition.

**Conclusions:**

This research supports and elaborates on several documented and accepted good practices for working with adolescents to improve nutrition knowledge and attitudes. Similar programs with key features such as co-design, suitable timing, curriculum, and exposure of programs by age group, addressing underlying structural issues, the involvement of gatekeepers and duty-bearers, and confidence-building can increase adolescent girls’ nutrition knowledge and attitudes.

## Teaser

A nutrition program co-designed, implemented, and monitored by adolescent girls demonstrates high potential for increasing girls’ knowledge and their self-confidence and capacity to improve nutrition knowledge of peers and community influencers.

## Introduction

Adolescence is a critical developmental stage, the second-largest period of growth, and when lifelong healthy habits can be formed (1, 2). Adolescent girls in low- and middle-income countries (LMICs) often face an increased risk of malnutrition due to low agency and gender inequality, including early marriage, early pregnancy, and inequitable intrahousehold food distribution. Adolescent girls have specific nutritional needs, particularly for iron, and are at an increased risk of iron deficiency anemia. Furthermore, the prevalence of overweight and obesity is increasing among adolescents in LMICs (3). There has been increasing interest in adolescent health and nutrition in recent years—for example, the inclusion of adolescents in global health strategies such as the Global Strategy for Women's, Children's, and Adolescents’ Health (2016–2030) (4).

Evidence-based interventions for improving adolescent nutrition include micronutrient supplementation and food fortification; school- or curriculum-based nutrition education; prevention of adolescent pregnancy and poor reproductive outcomes for adolescents who do become pregnant; and social and behavior change campaigns to improve health behaviors such as increasing physical activity and limiting processed-food intake (2). While evidence of effective actions to improve adolescent nutrition is growing, guidance on implementing these interventions is still limited. The WHO has published guidelines (2) outlining how to implement effective actions for improving adolescent nutrition. These guidelines identify research gaps, including the need to better understand how to strengthen adolescent participation, innovate delivery platforms, and evaluate services related to the promotion of healthy diets (2). Further, limited implementation research has been done to identify ways to successfully engage girls in designing and implementing nutrition programs intended to target them (2).

Nutrition International (NI), an organization that works worldwide to alleviate malnutrition among vulnerable populations, developed a program model in collaboration with the World Association of Girl Guides and Girl Scouts (WAGGGS) in early 2017 to improve adolescent nutrition. The Girl-Powered Nutrition (GPN) program was designed to influence the knowledge, attitudes, and behaviors of adolescent girls and their communities through a nutrition badge curriculum and advocacy activities in 4 target countries.

The program was structured around 2 components of awareness-raising/knowledge building through a nutrition badge activity pack and community mobilization/advocacy through community action initiatives and national/global campaigns. The activity pack was rolled out to leaders through a cascade model of training. The community mobilization and advocacy component was divided between 5 levels: at the individual level (to reach community members individually), at the community level (“Community Action Fund”), at the wider community level involving Girl Guide and Girl Scout units/troops/companies specifically identified to lead action groups (referred to as “Action Hubs”), at the national level (with advocacy campaigns in the Philippines, Tanzania, and Madagascar), and at the global level (with a global online advocacy campaign, and selected Girl Guides and Girl Scouts invited to attend global events on nutrition).

The 4 pilot countries—Madagascar, the Philippines, Sri Lanka, and Tanzania—were selected mainly due to their high anemia prevalence among women of reproductive age, as well as due to logistical considerations. This paper aims to illustrate adolescent girls’ experiences with the co-design and implementation of the pilot GPN program using the findings of an external formative evaluation. Additionally, it explores the potential for achieving program objectives of improving knowledge and attitudes on nutrition by providing nutrition messages to adolescent girls using a nontraditional platform.

## Methods

### Study design

This study qualitatively assessed the results of a formative evaluation of the GPN pilot program commissioned by NI and undertaken by Universalia Management Group (Universalia). This evaluation followed the Canadian Tri-Council Policy Statement on Ethical Conduct for Research Involving Humans (5) and, for Tanzania, the Guidelines of Ethics for Health Research in Tanzania (6). Ethics approval for this evaluation was granted in the Philippines by Eastern Visayas Health Research and Development Consortium, and an exemption for ethics (given the evaluative nature of the work) was granted in Madagascar from the Comité d'Ethique de la Recherche Biomédicale auprès du Ministère de la Santé Publique. Ethics approval in Canada was granted by the Veritas Independent Review Board. In Tanzania, ethics approval was granted by the National Institute for Medical Research, and a permit for the lead researcher (ED) to collect data in Tanzania was provided by the Tanzanian Commission for Science and Technology. A Data Transfer Agreement with the National Institute for Medical Research in Tanzania was also filed and approved for the evaluation data to be transferred to Canada for analysis.

Below, we briefly describe the GPN program and the methods for conducting this study, including data collection via interviews, focus groups, and document analysis, and a thematic analysis.

### Program overview

Briefly, the GPN 2-y pilot program was launched in early 2018 with the eventual aim of providing at-scale nutrition support to WAGGGS’ 10 million members worldwide. The evaluation focused on 3 of the 4 pilot program countries (Madagascar, the Philippines, Tanzania, and Sri Lanka). Due to bombings and the volatile security situation in Sri Lanka in 2019, it was decided not to complete that arm of the study. For the evaluation site selection, several criteria were considered: representation in each region, language diversity, large girl guide (GG) or girl scout (GS) population, and participation in advocacy campaigns. Sites within each country were selected based on the criteria of ensuring rural/urban representation, having a range of age groups, a mix of WAGGGS’ member organizations (MOs) (if applicable), and geographical representation.

The GPN program was structured around 2 components: *1*) nutrition education (curriculum) through a nutrition badge activity pack and *2*) community mobilization/advocacy through community action initiatives. In the early stages of the program, co-creation teams (CCTs) composed of girls from each age group were created in every target country. Two CCT workshops were conducted in each country. The first was on the curriculum itself and to select activities to be tested with units at the local level, and the second was primarily focused on community action activities and allowed time to discuss the logo, badge, and name of the program.

For the pilot program, the target for the first component was that 160,000 girls between the ages of 6 and 19 y would earn the badge. The activity pack was rolled out to leaders through a cascade model of training. The second component included access to funding for advocacy activities. The community mobilization and advocacy component was delivered in 5 ways: *1*) at the individual level with a target of 320,000 community members to be reached with each girl targeting 2 people in order to earn the badge; *2*) at the community level through small grants given to girls wanting to take further action (“Community Action Fund”); *3*) at the community level involving GG and GS units/troops/companies specifically identified and funded to lead action groups; *4*) at the national level with advocacy campaigns in the Philippines, Tanzania, and Madagascar; and *5*) at the global level with a global online campaign and selected GG/GS (advocacy champions) invited to attend global events on nutrition.

### Data collection

This study was a secondary analysis of the data collected as part of a formative evaluation of the GPN program. Universalia used a nonexperimental design and developed the evaluation methodology in consultation with NI. Universalia developed an evaluation matrix, including evaluation questions following the Organisation for Economic Co-operation and Development's Development Assistance Committee (7) standard evaluation criteria (relevance, effectiveness, efficiency, sustainability). A gender and equity dimension was included as a key cross-cutting criterion of the evaluation. The evaluation employed key informant interviews (KIIs) and focus group discussions (FGDs) to collect information from a wide range of stakeholders. Interview and focus group guides were developed based on the evaluation matrix created. Key documents were also gathered and analyzed as part of background information and for triangulation of data.

Data collection took place between September 2019 and February 2020. All participants signed consent forms; girls under 18 y signed assent forms, and their parents signed consent forms. KIIs took place with NI and WAGGGS staff based in London, United Kingdom, and Ottawa, Canada; local GG and GS associations; and advocacy champions (see **Table 1**). FGDs involved CCT members (adults and girls), 3 different age groups of GG and GS members (younger, middle, older age groups), adult leaders (guiders, *cheftaines* or guides), and community members (peers, siblings, parents, and community leaders). During KIIs and FGDs, respondents were asked about their role in the program, the CCT members’ involvement in the design of activities, and the relevance of the program considering local contexts. They were also asked about GPN's strengths and weaknesses, its unintended positive or negative impacts, the adequacy of resources versus expected outputs and outcomes, and their recommendations to ensure sustainability of results. A mix of census, criterion, and purposeful sampling (8) was used to select respondents. For staff in London and Ottawa, all those participating in the program were interviewed. For CCT and advocacy champions, all members were included. For other groups, the participants in each site were purposefully selected from a list of girls who were between the ages of 6 and 19 y and had participated in the program and hence could provide responses to the questions. In total, 70 people took part in KIIs, in-person, by Skype or by telephone, and 233 people took part in 50 FGDs (13 in the Philippines, 19 in Madagascar, and 18 in Tanzania). FGDs involved between 2 and 11 participants aged 6 to 70 y. GG and GS took part in focus groups with peers in their age group. The authors felt that saturation was reached because repeated themes emerged across groups and countries.

**TABLE 1:** Overview of evaluation consultations by country and type^1^

	Canada (Ottawa)2019	UK (London)2019	Philippines (Manila, Bulacan, Calbayog)	Madagascar (Antananarivo, Fianarantsoa, Ambositra)	Tanzania (Dar-Es-Salaam, Tanga, Lindi)	
			7–20 October 2019	21 October–10 November 2019	20 January–2 February 2020	Total per category
NI	6	—	2	0	1	9
WAGGGS	—	6	1	1	1	9
Local partners	—	—	4	7	4	15
Advocacy champions	—	—	4	3	7	14
Total interviews	6	6	11	11	13	47
Girls, younger age group	—	—	8	13	15	36
Girls, middle age group	—	—	21	15	15	51
Girls, older age group	—	—	15	18	13	46
Co-creation team	—	—	6	8	9	23
Boy Scouts attending GPN activities	—	—	—	3	—	3
Adult leaders	—	—	29	19	13	61
Community members	—	—	12	5	15	32
Regional Commissioner and Chairperson	—	—	—	—	4	4
Total focus groups	—	—	91	81	84	256
Total per country	6	6	102	92	97	303

^1^GPN, Girl Powered Nutrition; NI, Nutrition International; WAGGGS, World Association of Girl Guides and Girl Scouts.

All FGDs and KIIs were audio-recorded if participants provided consent. In the few cases where participants did not want to be recorded, detailed notes were taken, and the research team discussed these afterwards to ensure all details were captured. In accordance with the project's participatory approach, international consultants trained 14 selected young women in Tanzania, Madagascar, and the Philippines on data-collection methods and tools. These young women were responsible for facilitation and note-taking for the FGDs with GG, GS, and girl community members. National consultants were also hired in each country to conduct KIIs and FGDs in the region's local language, where applicable. In Canada and the United Kingdom, KIIs with NI and WAGGGS staff were conducted in English. In the Philippines, KIIs and FGDs were conducted in English, Tagalog, and Waray; in Madagascar, they were conducted in French and Malagasy; and in Tanzania, they were conducted in English and Swahili. The national consultants also transcribed all audio recordings and translated them into English, including some quotes presented in this paper. Data analysis in English and French was done by the authors (ED and SP). Everyone involved in data collection and analysis (e.g., facilitators, notetakers, national consultants) was trained in research ethics, including privacy and confidentiality, and signed confidentiality agreements.

Several strategies were used to ensure data quality, including ongoing meetings with WAGGGS and NI throughout the evaluation; in-depth training of local facilitators and consultants in the methods, ethics, and data collection; oversight of initial data collection in each country; and review of transcripts (with input from the national consultants as needed) to ensure accuracy and clarity. Social desirability bias was mitigated with peer facilitators for data collection where possible (e.g., adolescent girls), thorough training of data collectors, and emphasizing to participants that there were no right or wrong answers.

### Thematic analysis

Data were triangulated from different sources, thereby ensuring the validity of findings, conclusions, and recommendations presented. QSR International NVivo 12 for Mac (Canada, United Kingdom, Tanzania, Philippines) or Microsoft Excel (Madagascar) was used to organize, methodically code, aggregate, and triangulate qualitative data from interviews and focus groups, identifying recurrent conceptual themes by country and participant category. Authors ED (Canada, United Kingdom, Tanzania, Philippines) and SP (Madagascar) coded all the data. Data were first coded to nodes by question for each source. Following this, nodes were examined to identify patterns and common themes across sources and differences in responses between groups and to compare themes with the document review findings (9). The lead researchers (ED and SP) met to discuss themes emerging from the data analysis. Debriefs were held with the local consultants in each country (including OM in Tanzania) at the end of each field mission, including discussing themes and findings and integrating these into the results. The results were further validated in discussion with NI and WAGGGS staff.

## Results

The findings of the formative evaluation were organized into 6 themes: *1*) experiences with the co-design process; *2*) implementation fidelity and adequate time and intensity of program exposure for changes in knowledge and attitudes on nutrition; *3*) the effect of underlying structural issues, including poverty, societal norms, and gender inequality, on adolescents; *4*) suitable times for adolescent nutrition programming; *5*) relevant curriculum by age group; and *6*) self-confidence and skills, and adolescent engagement for influencing gatekeepers and duty-bearers (including community leaders, parents, and males) on nutrition.

### Theme 1: experiences with the co-design process

CCT members were viewed as playing a critical role in the first stages of the GPN program, from badge design to activities testing.

The analysis showed that key contributions from CCTs were to simplify wording and adapt curriculum guides to the local context, translate resources, test activities, provide examples of activities that could work well, share ideas, have moments of reflection on specific needs for each period of a woman's life (childhood, adolescence, adult, pregnant or lactating mother, elderly), and provide feedback and proposed adjustments on design and branding of the badge (form and colors) and the GPN program name.

WAGGGS and NI respondents noted that it was a new experience for NI to have a more bottom-up approach to empower girls to be involved from the start and that it was a relatively new experience for WAGGGS to have this level of girls’ involvement in the development of the program resources. The experience was felt to be generally participatory, although some WAGGGS staff reportedly felt that the girls could have been more involved in decision making for the curriculum development. Co-design was an essential process that the program sought to use throughout the design phase, and it did result in desirable outcomes both for the quality of the design and the girls' self-esteem. However, program staff did concede there was initially little know-how or experience around the co-design process and its parameters..

“We risked it being a bit lip service, or a bit just using them to get ideas—which is something that often happens with young people—but we did work to keep them involved—we did loads of best practice after—we had to be careful, and we were, in terms of the participation of young people—they weren't necessarily involved in decision making. It could have been a lot more decision making—we collected and respected their info and kept [them] up to date on decisions—but we made the decisions, instead of them making decisions.” —WAGGGS staff

“[It was a] learning process for us— co-creation doesn't mean doing exactly what they come up with. Us coming in as experts and professionals—[we know] this works and doesn't work, a number of names for the programs, some wouldn't work from branding or didn't sound right or were not in line with what WAGGGS would want.” —WAGGGS staff

While NI staff felt the role for the CCTs was more one of validation of the curriculum than of participation in the development of the curriculum per se, some CCT members felt their ideas, feedback, and inputs were taken into account, incorporated, and valued by WAGGGS during finalization of the activity pack, and that the workshops were participatory. WAGGGS staff also mentioned that they made decisions based on inputs from the girls, as shown in the following:

“We are so happy because most of our inputs were included in the activity pack and even with other countries.” —CCT member, the Philippines

“It was full of participation because they [facilitators] were with us, they would hear our contributions, it was participatory.” — CCT member, Tanzania

Despite these testimonies, the evaluation team did not find any concrete evidence to show that CCTs’ feedback and inputs were adequately incorporated in the curriculum, and some CCT members themselves were unable to link the final changes in the curriculum with what they had discussed. Some also noted that they never really knew if their contributions had been taken into account in the final version of the badge and curriculum.

“We gave a lot of suggestions as CCT members. While we cannot distinguish them in the final version [of the curriculum], they were integrated in the whole process.” —CCT member, Madagascar

However, WAGGGS provided documents in which the curriculum writer collated feedback from all CCT workshops and pilot testing. These documents include evidence of WAGGGS’ effort to include the girls’ feedback and inputs in the program's design.

Among challenges mentioned by respondents, the issue of getting girls comfortable to speak during workshops, and to have the confidence to offer their opinions, was raised, especially in the context of gathering adults with young girls (in Madagascar, for example, the youngest CCT members were aged 10 y). Some girls felt that there was a lot of new information provided during the workshops, and they did not feel adequately prepared to be part of the CCT as limited information was provided to them ahead of time.

In a few cases, the CCT members did not know the name of the program and said they were still waiting for feedback from WAGGGS headquarters, despite the program already having been named. WAGGGS mentioned a contract for CCT members; however, this document was not provided to the evaluation team and some CCT members explained there was no documentation to formalize the existence of the CCT. These examples likely reveal challenges with the communication loop and feedback mechanism. Finally, some CCT members could not remember much of what happened during these workshops, which occurred almost 2 y before.

The CCT helped with ensuring the adaptation to the local context.

“The curriculum for the GPN (program) is country-specific. The CCT was vital to make it more relevant for the context. Binalot is a Filipino term. The CCT was kind of vital to make it reliable. The Program Manager said something about a sandwich—we had to change some terms from English to our dialect—this was localization.” — CCT member, the Philippines

Attempts were made to ensure that the curriculum addressed local nutrition myths and included common foods in each country. During testing of the curriculum with CCT members, issues with country-specific contexts (e.g., lack of familiarity with rice in Tanzania) were mentioned as having been addressed.

“The module information was developed from the Girl Scouts of the Philippines (GSP) girls themselves—they know about the cultural sensitivities of different regions, and the food preferences of teenagers, and peer to peer influences.” — GPN staff/senior volunteer, the Philippines

However, Malagasy CCT members explained that they flagged elements not adapted to their region, but their inputs were not considered in the curriculum final version due to lack of time. Some words were changed to reflect food habits; however, various examples from the Philippines and Sri Lanka were kept to inform the girls about different cultures. Ensuring sufficient time to incorporate feedback from adolescents was a key learning from the evaluation.

In the Philippines, respondents generally felt the curriculum had been localized (e.g., using local food examples, locally relevant food preparation, culturally relevant activities). It also seemed that the global curriculum used a few examples from the Philippines, such as the “*Binalot* Surprise.”

“Two activities in the pack—*Binalot* Surprise and Unwrap the Surprise—are in the global curriculum, but they came from the Philippines.” — GPN staff/senior volunteer, the Philippines

In the Philippines and Tanzania, it was felt the curriculum aligned with each country's realities in terms of what food types are available and what is affordable.

“I think the guideline is normal and it considers our environment because people can plant vegetables like spinach and get nutrients. This shows that it is not for rich people. It fits everyone.” —Adult leader, Tanzania

However, in Tanzania, some examples in the curriculum were also considered too “European”; therefore, adaptations were made.

In Madagascar, opinions were also mixed, as several respondents explained that the country's diversity, in terms of local cultural, social, and economic specificities, was not sufficiently considered before implementation.

“It is not that we did not like it, but it was difficult to transfer knowledge. All examples given [in the curriculum] were examples from abroad, and it was not possible to relate to what is here. The best would have been to give examples from here. It would have been easier for participants to understand.” —Girl, older age group, Madagascar

Last, some CCT members reported that aligning activities with age categories during the curriculum development was a challenge. The testing phase for the curriculum development helped to adjust this.

### Theme 2: implementation fidelity and adequate time and intensity of program exposure for changes in knowledge and attitudes on nutrition

While GG and GS (girls) seem to have understood many of the key messages on nutrition and improved their knowledge and attitudes to make healthy choices, misconceptions about nutrition messages remain.

Girls identified messaging that they had learned to date on nutrition, including planning meals, adequate nutrition such as eating more vegetables and fruit, having colorful plates, looking at food packaging or food labels (Philippines and Madagascar), and the importance of iron, carbohydrates, calcium, vitamins, and protein. They also appeared to be more conscious about the importance of avoiding industrial or processed food and reducing sugar, salt, and oil for a healthy diet, particularly in Madagascar and the Philippines.

Many girls explained that being healthy is not only about how much you eat but about the quality of your diet and your lifestyle as a whole: drinking water, exercising, consulting medical services when needed, applying basic hygiene measures (washing hands, rinsing fruits and vegetables), and getting adequate sleep.

“We should eat healthy foods for the bones, we should exercise, drink more water, we should sleep 8–10 hours a day.” —Girls, younger age group, the Philippines

“Before eating, I will think first if it is healthy and its effects on me,” —Girl, middle age group, the Philippines

Many girls noted that they now better understand girl-specific needs and what type of food they need depending on their age and life stage.

“For example, a mother that is pregnant but not healthy—as a result her baby is not healthy. Also that's why a girl needs specific nutrition.” — Girl, older age group, the Philippines

Girls mentioned iron as an important nutrient for adolescent girls once menstruation begins and for women in general.

“A girl is supposed to eat food that has iron because when you start [your] period, you lose a lot of blood, so you need to eat fish and leafy greens to help recover it.” — Girl, middle age group, Tanzania

Calcium was also mentioned for women in general, and in some cases, for older women specifically. Girls also mentioned that they learned how to make healthy meals that do not cost too much.

“I have learned how to make a rainbow plate in an affordable way without using too much money.” —Girl, older age group, Tanzania

However, there were girls in the FGDs who could not identify any key messages. FGDs also confirmed some misunderstanding of nutrition messages, although many had not yet completed the activity pack. Girls sometimes gave incorrect information or were confused about nutrition messages—for example, confusing the micronutrients iron and iodine, not being able to list foods containing protein, or believing myths about nutrition.

“A sick person cannot eat what you are eating. For example, you are eating dairy products like yogurts, milk, cheese, and the sick person cannot eat these.” —Girl, younger age group, Madagascar

“If my mother cooks a hotdog, she should add eggs so at least there will be protein.” —Girl, middle age group, the Philippines

Moreover, the advocacy champions and CCT members asked for additional nutrition information and education to help them with their role in the communities. This included the wish for more time for training and more general knowledge on nutrition, statistics, and practical applications.

“Maybe more seminars to be aware of recent statistics on nutrition and the Philippines. I am not very knowledgeable—[on] what to eat or what to advise on healthy standards.” —Advocacy champion, the Philippines

“You are being led through very fast because you only have two days, after that, you have already studied [been through the training], after that you start thinking what was this again, or maybe you are not doing much practice, you find that you are trained more with words, but you don't practice, it becomes like, it's not enough, you find it's as if you have too many words but no practice, it becomes something like that.” —Advocacy champion, Tanzania

“But they [CCT] didn't have background information—some felt that it was like being put in the deep end—they were hearing information for the first time. This was a lot of information for those girls—and they are really young—and they were hearing this for the first time.” —GPN staff/senior volunteer, Tanzania

“The training itself was sufficient, however more supervisory support would be useful. Gaining confidence through practice to manage our stress and dare to speak to decision-makers.” —Advocacy champion, Madagascar

### Theme 3: the effect of underlying structural issues, including poverty, societal norms, and gender inequality, on adolescents

While some barriers were addressed through the GPN program, many challenges remain, including cultural norms, poverty, and patriarchy.

While respondents felt that they learned how to prepare low-cost, nutritious meals, poverty remains an obstacle to implementing the learnings and reaching others.

“I have a friend who gets sick a lot, so I always ask them ‘Do you eat proper nutrition?’ She says ‘I try, but money isn't enough,’ so I tell her how to create a complete meal with a little money.” —Girl, middle age group, Tanzania

The activity pack focused on individual dietary behavior change and not as much on broader determinants of nutrition, such as poverty and gender.

Cultural norms and myths related to nutrition remain and should be addressed.

“Some community members believe that if you eat leafy greens or ‘dagaa’ [type of sardines], then you are living a low life [it is a sign that you are struggling financially]. Because they don't know these foods contain special nutrients that are really important.” —Adult leader, Tanzania

This includes involving men and boys in the program to achieve broader, longer-lasting change, given their household decision-making roles.

“Also, the decision of foods—in most regional areas . . . the father decides what to eat—so he buys what he wants, not what others want.” —GPN staff/senior volunteer, the Philippines

“Certain communities still don't want girls to eat certain meats or parts of animals such as liver, but this can impact the girl, because she might not get enough protein, and won't be able to grow properly.” —Girl, middle age group, Tanzania

While the GPN program was aligned with NI's gender equality focus and was designed to be inclusive, in practice, the GG and GS movement might not always be inclusive of the most vulnerable girls (e.g., the poorest or those out-of-school). It was revealed that GG and GS participants might be more representative of the middle class, given the costs of registration, uniforms, and the opportunity cost of participating (less time to work or help at home). Staff generally felt that this was not an issue for the GPN work, stating that the movement is inclusive. Others (e.g., community members who are parents of the girls) mentioned that cost (e.g., for the uniform) was an issue. The GPN program may have reached those with more money and girls in school (in Tanzania and the Philippines, where the GG and GS are school-based).

“Some of the children are missed here, those who don't attend schools. And there are a lot of them.” —Community member, Tanzania

Ways to improve access to GSP for more vulnerable girls were being considered.

“Gap in financial support, how do we reduce inequalities so GSP is more inclusive and accessible, and not just middle class.” —Advocacy champion, the Philippines

In Madagascar, some stakeholders perceived that the scouting movement reaches all social classes: girls from rural and urban areas and with more or less resources.

“It is not very expensive to be a scout in Madagascar.” —GPN staff/senior volunteer, Madagascar

In Tanzania, it was noted that the curriculum provided opportunities to teach people to eat nutritiously even with low income; however, poverty was still noted as a significant barrier to healthy eating.

“Things that stop me from eating healthy food is poverty.” —Girl, younger age group, Tanzania

Similar issues were identified in the Philippines.

Gender considerations are covered in the program documentation, and there was a strong focus on the inclusion and participation of girls in the curriculum development and addressing girls’ specific nutrition needs and risks, in alignment with NI's priorities. NI also provided guidance on the inclusion of gender mainstreaming in the curriculum. However, gaps in the curriculum were noted in terms of systematic upstream determinants of health-related gender and poverty issues, such as child marriage and how food is distributed.

“But in terms of child marriage, poverty, gender allocation of food—we probably haven't looked at these determinants—we do address some in the advocacy campaigns—but really it was focused on individual behavior change.” —WAGGGS staff

There was a high level of interest in involving boys in the GPN program. Many respondents regretted that boys were not involved in the program, particularly given the role males play in many countries in terms of household decision making, including nutrition.

“Boys also should learn, because they are also the fathers of tomorrow. If it happens, they end up with a woman who doesn't know about this nutrition stuff, he can step in and educate his family.” —Community member, the Philippines

Leaders also noted that boys wanted to learn about nutrition in the schools.

The program may inadvertently be reinforcing traditional roles of women and girls in society instead of conveying a message of empowerment. KIIs and FGDs in all countries revealed some lack of clarity around the concept of “girls’ sex- and gender-specific needs” and what NI's and WAGGGS’ expectations were in terms of girls’ empowerment. While one of the main program objectives was to “promote girl-led change” “by providing knowledge to girls and young women in communities” (10), on the ground the program tended to reinforce the traditional role of the female as wife and mother. A clear message was that it is important to teach girls about nutrition because of their role as mothers in the future. This message far outweighed any message of girls’ empowerment.

“I also remembered during the training that it was discussed because as they are still young, their mind should be more open when it comes to nutrition. They will be mothers soon, so, at their young age, it not just for themselves. Because it will come from them to the extent that they will be the ones cooking for their family and other girls”; “And they will prepare the food in the future.” —Adult leaders, the Philippines

“It will also prepare them for their future life as mothers.” —Girl, older age group, Madagascar

“When girls eat properly and are healthy, then even during labor there are less complications. They are also strong enough to push during labor. It's easier for them to give birth.” —Community member, Tanzania

“Our curriculum goes much deeper, at the end of the training, by the time the girl gets her badge, she has become a great mother, and not just an average mother.” —GPN staff/senior volunteer, the Philippines

“From my point of view, I think it is better that [she learns] now that she is still a young girl and single, that she already has knowledge on nutrition because she is going to have a family and run a family later.” —Community member, Madagascar

Even discussions on the need to shift away from boys eating more (or better) foods than girls are framed in the context of girls’ role as mothers.

“I'd educate the members to let go of beliefs that prioritize boys over girls because you will find when serving food some say give him the meat you know he is a man, so I was trying to educate them that the girl is first then everyone else after. That this girl needs good nutrition from when she is growing to later when she becomes a mother herself. If she doesn't get this, then her offspring will also be affected. So, in providing for the girl, we are providing for the entire society.” —Adult leader

Another issue in Madagascar was related to the popular beliefs around women's bodies and girls internalizing that healthy nutrition will help attain specific beauty standards around size.

“They will also be able to balance their diet to not be too big for their age”; “They have to know what a healthy diet is to not be too tall or too small, too big or too thin, for their age.” —Girls, older age group, Madagascar

### Theme 4: suitable times for adolescent nutrition programming

Overall, evidence demonstrates that GPN activities were not consistently implemented at suitable times for the girls, sometimes interfering with the school calendar, schoolwork, household chores, and other GG and GS activities. This situation is likely due to a time-consuming program, which required girls to be available in a short period, and ambitious reach targets given the limited time frame for implementing the GPN program.

Time slots allocated to GPN activities were inconsistent from one country and group to another because they were based on when the GGs and GSs typically met in each locality. In most groups in Madagascar, GPN program activities were organized twice a month. In general, groups devoted 1 h out of their 2-h meetings to the program, with meetings taking place mostly on Sunday afternoons. GPN activities were also implemented during camps, especially the practical activities. In the Philippines and Tanzania, where GG and GS programs are school-based, reaching girls at school appeared to be effective. Generally, girls interviewed in Tanzania found they had time to participate, as it was typically 1 h per week, after school, on weekends, or at a set time.

“That age group 6–19, it's an age group that the child is at school, so it has become easier for us, because we are with them all day, from morning to evening, it's an age group that the girls are studying in school, so it easier for us to get to them.” —Adult leader, Tanzania

In the Philippines, adult leaders mentioned that they instructed the girls after class, including during lunch breaks, and excused them from class to participate (for the latter, in high school in particular). The leaders fit these sessions in where they could to implement the badge curriculum and were finding this a challenge to maintain.

“In our case ma'am, every Friday, I excuse them from their classes. So sometimes they leave their classes just to attend my GPN activities. And even Saturdays just so that I can continue with it [GPN activity] for at least 2 days.” —Adult leaders, the Philippines

In all countries, adult leaders had to compete with the girls’ school curriculum and calendar. In Madagascar, for example, the GPN program started in the middle of the school year (March/April for most groups) and most respondents mentioned that it would have been easier to implement the program's time-consuming activities during school holidays. For others, especially with school-based GG and GS activities, implementation during school holidays was very difficult because many girls (and boys) live in remote areas, far from their schools, and cannot attend activities outside of school time.

Many adult leaders reported that attendance at GPN activities was more a question of availability than motivation for acquiring the badge. Causes of absenteeism were related to parents’ priorities, the distance between home and the meeting place, school holidays, family events, and exam periods (especially for older girls). As GPN implementation began midyear, adult leaders in Madagascar were not ready to integrate GPN activities in their yearly program. In some cases, this resulted in prioritizing GPN activities over other badges or delaying GPN activities to finalize other badges already started, especially other badges funded by WAGGGS.

The mitigation strategy adopted by adult leaders was to tailor the program to girls’ availabilities, divide groups between the ones attending regularly and the others, encourage girls to share information with those who missed activities, and fit in GPN sessions where possible.

“To improve students’ performance [in school], they came up with the plan that the children need to stay late after school, up to 6 p.m. in the evening. And the parent should contribute so the children can get food. That creates a challenge because we do not get the time to do guide activities. But the guiders try their level [very] best during breaks for twenty or forty minutes to conduct nutrition activities because these activities are outside the school system. At the same time, our goals are to make sure that the girls reach their dreams, and we want them to attend normal school activities. So, it is challenging sometimes.” —GPN staff/senior volunteer, Tanzania

In the Philippines, the girls mentioned that they had to self-study and catch up on their own time for any school days they missed working on the badge curriculum. Some GSs noted it took much effort on their part. In some cases, it was worth it because they were learning and enjoyed the activities. However, it caused some stress on the girls at the junior and senior levels as they had to make decisions based on their priorities.

“Sometimes I become irritable when I have projects that need to be rushed for submission, and then I have to attend some activity as part of my obligation since it's my responsibility and obligation to attend the activity then I have no choice but to attend to it.” —Girl, older age group, the Philippines

“Facilitator: What problems, if any, do you face in taking part during these times? Respondent D: We panicked what to do first. Respondent H: Same, because sometimes it has the same schedule as our academics. Facilitator: Then what do you do? Respondent E: If it is academics, we are excused from the class. I weigh things which are more important.” —Girls, middle age group, the Philippines

In Tanzania, a similar observation emerged, as some girls noted they had schoolwork and chores and that it was sometimes challenging to balance this work, including finding time to reach the community with messages during their spare time.

It was also mentioned that because GPN sessions were held after class, some girls were hungry when attending scouting activities, and some would instead go home and miss the session to eat.

“There are challenges, for example, feeling hungry during the time for Girl Guides, you might really want to go but you can't, so you end up going home.” —Girls, younger age group, Tanzania

Finally, a few community members also mentioned the potential challenge of interfering with girls’ chores at home.

“Also when it comes to chores at home, it's another challenge, because for those who have understood the importance we can understand that is why we are even here, but for some parents who don't know better they think that in Girl Guides they think the girls are going to waste time, so they'd rather the child remain home to wash dishes instead of going to a meeting.” —Community member, Tanzania

### Theme 5: relevant curriculum by age group

Activities were considered to be designed according to age groups (younger, middle, and older) and considered to be appropriately adapted to GGs and GSs. For example, in Tanzania, it was felt that activities were specific to each age group's capabilities, less in-depth for the younger groups and more in-depth for older groups. No major concerns were raised in terms of applicability by age group.

“The activity guide for obtaining the badge is developed taking into account the absorption capacity of each age category as well as the specific nutritional needs of each age.” —Adult leader, Madagascar

“If you look at the books [curriculum], the first is very small, the second one is a little bit bigger, and the last one is the biggest, but the key messages of the books are the same. So, the difference is in the depth of what they are learning. For example, the first group is taught how to mention food, and the other group is trained how to prepare [food].” —GPN staff/senior volunteer, Tanzania

Since girls could relate to the badge curriculum appropriate for their age group, they retained the information. The activities that the girls participated in from the activity pack were delivered by a trained leader and conducted in a fun and playful way through games and songs to teach the messages. This was viewed as helpful to keep the girls’ focus (especially the younger group), their interest, and enhance their understanding.

“If the guider is very good, it is a lot of activities and a few words, and a few games to make sure the child is active, and when they go to reach the community, it won't be a very hard job for her.” —GPN staff/senior volunteer, Tanzania

“We try to be recreational in order to arouse their interest and to carry out outdoor activities.” —Adult leader, Madagascar

While it was felt that the messages were easier to understand for older girls (16–19 y), most girls enrolled in GG and GS activities were in younger or middle age groups. For example, there were no older groups in some Madagascar regions as adolescents reportedly do not have time or interest in GG and GS activities. The same observation was made in the Philippines, where there were limited numbers of girls in GSP who were 16–19 y of age. Targets were consequently challenging to meet for these age groups in these regions.

### Theme 6: self-confidence and skills, and adolescent engagement for influencing gatekeepers and duty-bearers (including community leaders, parents, and males) on nutrition

The reach to communities was not as high as had been planned. Community members (including parents) reported that they were not sufficiently informed of the GPN program, what their daughters were trained on, or the objectives. Some community leaders in Tanzania felt that, given the bottom-up approach used for the GPN program, they, as leaders, were not adequately involved.

Several respondents also mentioned that they would have liked to have seen parents more involved in the program, given their interest and the necessity of their involvement to ensure buy-in and sustainability. Respondents felt that knowledge would be more effectively maintained if parents were more involved in the program and communication was stronger between those implementing the GPN program and parents themselves.

To obtain the badge, each girl had to share nutrition messages based on what they learned with 2 non-GG or non-GS people. Messages mainly included very general statements on the importance of eating a balanced and diverse diet and growing and eating leafy green vegetables, food groups, and other generic messages on “proper nutrition.” A few girls also mentioned having provided more specific information to peers, such as the importance of iron intake and menstruation.

“The children are now advising parents that they should not cook or prepare the food without vegetables and fruits.” —Adult leader, Tanzania

“Yes, I educated girls my age about what happens during menstruation, and the importance of the mineral iron.” —Girl, older age group, Tanzania

Various comments regarding influencing community members were made during interviews and FGDs. Activity packs for middle and older girls gave examples of community members that the girls could reach out to. Despite this, it appears that community members reached were primarily people close to them. The main reason given was that it was easier for young girls to talk to and influence women and girls within their close circles than to reach “influencers” or “authorities.” Respondents mainly mentioned girls and women close to them (e.g., mothers, sisters), but less often girl peers at school. Fathers and brothers were rarely mentioned (although “parents” were sometimes identified), raising the question of men's inclusion in the discussion on nutrition.

Adult leaders during KIIs and FGDs raised the issue that it was unclear how to verify the extent to which the girls had reached community members and to measure the quality of their messages (e.g., accuracy and comprehensiveness).

Many respondents mentioned that girls were more self-confident and had acquired skills to influence their family, especially sisters and mothers, and friends, with many participating in nutrition discussions. Respondents mentioned behavior changes observed during camps, and parents attested to changes at home. During camps, girls who attended GPN activities were proud to share their new knowledge with other girls. They demonstrated creativity in cooking healthy with limited quantities of food or budgets. Further, the girls were curious to find out more; they queried food packaging, checked ingredient lists, and questioned what was said on TV and radio, especially advertisements.

“I have learned that proper nutrition is important. Also it has given me confidence to teach other people because in the beginning, I was afraid to talk to people about it, but now that I know more, I am more confident.” —Girl, middle age group, Tanzania

Advocacy champions and those working on a badge noted that the girls had started to influence others who were not GGs and GSs, including their family members or peers.

“In terms of building confidence, to all the girls, first of all, once you are involved in Girl Guides, they help us build self-confidence, if you just pass through Girl Guides, 2–3 years, it is must that you will be confident enough to speak, to stand and speak, also even in those events that we are telling you about like thinking day, this past week we had Nutrition Bonanza, the girls were giving a demonstration. There was a small child explaining to the guests of honour, she was like speaking that this is what we should eat, and they were showing a play that this is our reality, but this is what we should do, and the people who were given a chance to talk, spoke, so confidence is there.” —Advocacy champion, Tanzania

Some girls were quite frustrated, explaining that they did not always feel they were taken seriously by adults in their communities, particularly those outside of their families. It was questionable whether girls had enough influence to convince other community members (e.g., males, older adults) about nutrition and whether community members would be willing to listen.

“I think the coordinators from the project need to have a good connection with community leaders and not leave the responsibility to the children to talk to the community, because the children have a lot of responsibilities, and also people in the community might not listen to children. Instead, using community leaders, they could have talked directly with the community.” —Community member, Tanzania

“Facilitator: why do you think you can't influence your barangay [smallest administrative division in the Philippines]? Respondent A: Because I'm still young.” —Girl, middle age group, the Philippines

Several girls also emphasized that local and national authorities should disseminate nutrition messages to the population.

“The last time we did an awareness activity for the GPN, people tend to take what we were saying for a joke.” —Girl, older age group, Madagascar

Despite this context, a few girls mentioned positive changes in their close circles. Some explained their families were making some changes, such as adding vegetables to meals.

Overall, it appears that GGs and GSs had many ideas and were motivated, but limited messaging to communities had been done at the time of the evaluation and community members may not have been responsive to the girls’ initiatives without further support due to the perceived lack of credibility of adolescents.

WAGGGS staff reported that they found the advocacy champions had increased their confidence, including in public speaking following training.

“And the end of the weekend they were doing presentations, public speaking, speaking in front of regional commissioners—it is just amazing—and after they did their presentations—did off the cuff and said thank you—and they said I found my voice, can't wait to get started—evidence that confidence increased during a short time.” —WAGGGS staff

The global advocacy activities that were planned were carried out, including advocacy champions attending Women Deliver 2019, the Commission on the Status of Women conference in 2019, and a Washington, DC, meeting in 2018 on creating campaigns in nutrition. This resulted in girls being confident to speak and provide leadership in nutrition. One success story was mentioned explicitly during interviews: in December 2018, a Malagasy advocacy champion took part in the Global Citizen Festival in South Africa, during which she met with World Food Programme and FAO representatives, as well as the Madagascar Secretary-General. She promoted a petition to increase the Malagasy government's budget dedicated to nutrition. According to several respondents, her advocacy action largely contributed to increasing the national nutrition budget from 1% to 3% in the following months. In addition, through their active presence on social media, advocacy champions successfully reached their peers, whether or not they were part of the GGs or GSs.

In interviews and FGDs, the advocacy champions revealed that they generally would have liked more support from the MOs in implementing the work and more support from global-level WAGGGS on understanding advocacy generally.

“Maybe they can conduct more training for girl facilitators—we were not given proper training, just webinars—although we believe that launch was successful.” —Advocacy champion, the Philippines

## Discussion

Limited global evidence on effectively engaging adolescents in nutrition programming through existing platforms is currently available. Earlier research looked specifically at health promotion programs in Scouting and suggested that health and nutrition knowledge can be improved through Scout-level messaging, but little is known about applying this messaging into practice at home, school, or in community settings (11). The GPN program formative evaluation reinforced already known good practices for working with adolescents to improve nutrition and health, sometimes with additional critical considerations, and offered some emerging good practices (see a summary of lessons learned in **Table 2**).

**TABLE 2:** Summary of lessons learned for working with adolescent girls on improved nutrition knowledge and attitudes through programming

Lesson 1: Clearly define and allocate sufficient time for adolescent girls’ involvement in the development of programs.	The involvement of adolescents in the design, implementation and evaluation of the nutrition programming that targets them is essential for meaningful uptake. Sufficient time and respect must be given to the co-design process, including clearly defining adolescents’ roles, ensuring transparency and clear communication, and managing adolescents’ expectations.
Lesson 2: Ensure implementation fidelity and adequate time and intensity of program exposure for changes in knowledge and attitudes on nutrition.	Program fidelity is of particular importance when using a cascade training model across global geographies. Program staff need sufficient training to feel confident in the nutrition information they are imparting, and adolescents require some supportive supervision when implementing program activities. Using evidence to define and then ensure adequate program exposure may help adolescents of varying ages and stages improve knowledge and attitudes on nutrition.
Lesson 3: Address underlying structural issues which have a unique effect on adolescents, including poverty, societal norms, and gender inequality.	Program curriculum and activities should be tailored to account for, or address where possible, the underlying structural issues that may negatively impact adolescent nutrition. Particularly, harmful norms around girls’ and women's traditional roles as vectors for childbearing and raising must be addressed; girls and women have the right to good nutrition for themselves and their own lives regardless of their roles as potential future mothers.
Lesson 4: Ensure program activities take place during suitable times for adolescent girls.	Ensuring programmers have a good understanding of adolescents’ schedules and competing demands, including expectations from gatekeepers such as teachers and parents, can help implementers choose suitable times for nutrition program activities for adolescents according to their age and stage.
Lesson 5: Reach girl children and adolescent girls with relevant curriculum by age group.	Adolescence, defined as 10–19 y, is a relatively long stage that includes considerable growth and change. Linking nutrition program strategies, curriculum, and activities to relevant adolescent age and stage are crucial for effective uptake.
Lesson 6: Increase self-confidence and skills and engage adolescents in influencing their gatekeepers and duty-bearers (including community leaders, parents, and males) on nutrition.	Adolescents are largely still reliant on their parents or other gatekeepers for their nutrition. Improving adolescent nutrition knowledge and attitudes through programming may be more successful when relevant gatekeepers and duty-bearers are aware and involved. As males often hold higher positions of power and influence in low- and middle-income country communities, involving adolescent boys in nutrition programming, which includes elements of gender equality, as well as engaging older males, may improve males’ ability or desire to act as allies for improved adolescent nutrition. Nutrition programs should include specific actions to support and improve girls’ self-confidence and advocacy skills to influence others on nutrition; this is particularly true for those in power, including influencing males or community members outside of girls’ close social and familial circles.

Addressing the underlying ecological and psychosocial barriers to nutrition is essential for, but not unique to, working with adolescents (12), and our study findings supported this. In their systematic review, Levy et al. (13) outline that broader norm change (i.e., beyond individual power of adolescent beneficiaries) is essential to effectively reach adolescents to improve gender equity and health, which is best achieved through multisectoral action and multistakeholder involvement. The present formative evaluation results demonstrate that, without addressing structural barriers and underlying causes of malnutrition, programs aiming to improve adolescents’ nutrition knowledge and dietary practices will likely not reach their full potential. The evaluation highlighted the need for enhanced gender training at all program implementation levels—training that would ideally be structured and tailored based on a sex- and gender-based analysis for each program geography. Any curriculum and training should include additional information to enhance participants’ understanding of gender equality and linkages to nutrition. This will be important to ensure that girl empowerment is supported more deeply and does not inadvertently reinforce gender stereotypes of women's primary roles as mothers, wives, and caretakers.

Addressing underlying barriers to nutrition with an adolescent lens means, at least in part, drawing on policies, strategies, and curricula with adolescents as a specific demographic with unique health needs (14, 15). Evidence-informed interventions should be targeted to gatekeepers and duty-bearers of adolescents (such as family members, teachers, and community leaders) as well as adolescents themselves (12). Building strong relationships and mutual trust with local partners, including local government, schools, parents, and communities, was viewed as a good practice throughout the GPN program. The GPN evaluation confirms the need to develop contextually and age- and stage-relevant curricula and engage gatekeepers and duty-bearers, such as families and the wider community, particularly males, to work with adolescents effectively. Given the important role that parents play in adolescent diets and the traditional role of males as decision-makers in many countries, consideration should be given to a broader engagement of parents and boys and men in adolescent nutrition and health programs.

It is well accepted that, to engage adolescents effectively, they must take an active role in their programming. Adolescents’ participation is 1 of 8 key standards outlined in the WHO's (15) *Global Standards for Quality Health Care Services for Adolescents*. The Standards recommend that adolescents be involved in the planning, monitoring, and evaluation of health services and decisions regarding their care, as well as in appropriate aspects of service provision. The GPN evaluation results support these principles when utilizing a community platform; the girl-led co-design, cascade model of implementation, and even involving girls in the evaluation process positively contributed to program uptake and perceived effectiveness.

Important considerations around the co-design process were identified through the evaluation. Clearly defining roles and expectations around adolescents’ input and how it will be used is important for both the adolescents and the program staff. It appears important for the adolescents to see how their inputs are used, so a plan to deliver this feedback should be set and communicated early in the co-design process. It also appears necessary for program staff to ensure the co-design process is truly collaborative and transparent. Program staff should be clear and intentional about the meaningful inclusion of adolescents’ input while managing expectations around the extent to which input may be incorporated into the final design and the mechanisms through which the co-design process will result in final decisions. Finally, ensuring adequate time for the co-design process is essential.

Based on our review of existing literature, 2 themes from this evaluation may offer new ideas or lessons learned for good practice in working with adolescents on nutrition and health programming. First, implementation fidelity should be monitored and adequate program dose for changes in knowledge and attitudes on nutrition should be ensured. The evaluation also identified some remaining misconceptions about nutrition, which may indicate insufficient program dose, inadequate exposure time to date, or lack of fidelity in transferring knowledge during the cascade model of training from international to national to regional levels. It was beyond the scope of the evaluation to assess the training curriculum (original and translated) against program objectives and results or the effectiveness of training.

The evaluation also found that engaging adolescents in reaching community members with nutrition messages and increasing self-confidence and skills to influence others on nutrition were important good practices in working with adolescents in nutrition and health programming. Results from the evaluation suggest that, despite reported increases in curiosity, confidence, and acquired skills in influence and advocacy, girls were only comfortable or able to reach other girls or women already included in their close social or family circles. Expectations around adolescents’ ability to influence community members will need to be adjusted by programmers based on age and life stage of the adolescent. Good practices for programmers for developing contextually appropriate and realistic expectations, as well as potentially legitimate and necessary limitations, for adolescent influence require additional research and guidance. Further opportunity exists to explore how girls’ influence, access (including the ability to utilize platforms like social media), and confidence to reach others outside their immediate social and family circles with nutrition messaging differ by age group and geography. Age- and stage-appropriate actions to help build girls’ confidence for advocacy activities around nutrition could be identified. As some evidence suggests that adolescents prefer nutrition messaging delivered by experts (16), ensuring that the nutrition messaging with which girls are reaching their communities is technically aligned with messaging from local and global experts is vital for supporting their confidence. In all, the GPN program evaluation revealed some powerful examples of girls harnessing improved confidence and skills in nutrition advocacy, and even some early successes in influencing nutrition policy and budget at the national level.

It is hoped that this research will contribute to the knowledge base around effectively working with adolescents to improve their nutrition and health. Adolescence (10–19 y) is when lifelong healthy habits can be developed and when nutritional demands are greater than at any other stage of life. Nutrition has a profound impact on the current and future health of adolescents, so it is critical to get programming right. Integrated programs that aim to improve adolescent agency and involve multiple stakeholders including gatekeepers and duty-bearers can lead to sustainable health and well-being improvements. Sustainability is further enhanced by challenging often harmful norms and behaviors related to gender at an early age and the gendered systems, all of which can ultimately impact nutrition. Reaching adolescent girls is important to support their agency as individuals and avoid girls’ demarcation as solely being future mothers. More research needs to be done on effectively tailoring nutrition programs to support adolescents’ unique needs, including how best to ensure adolescent participation in all stages of health programming.

### Limitations

This study had limitations related to design, implementation, and transferability. With regard to the design and implementation of the evaluation, choosing an ideal time for a formative evaluation to ensure that enough activities have been implemented while still ensuring that people can accurately recount their experiences was a challenge. Each country and region were at different implementation stages when data were collected (see Table 1 with field visit dates in each country). In Madagascar and the Philippines, the evaluators felt that the implementation of program activities might have been “sped up” to accommodate the evaluation. In all 3 countries, there had been limited implementation of many program elements by the time data collection occurred. In some cases, time may have passed between the implementation of an activity and the evaluation, which may challenge recall. In addition, social desirability bias could be a concern, but this was mitigated by using peer facilitators for data collection where possible (e.g., adolescent girls), having thorough training of data collectors, and emphasizing to participants that there were no right or wrong answers. Finally, other programs related to nutrition made distinguishing between the GPN program and other programs difficult for some. In interviews and focus groups, people were asked to be as clear as possible when an activity took place to understand if it was a GPN-related activity or another nutrition activity (e.g., annual nutrition month events). Triangulation of data across multiple sources and methods also helped to distinguish programs where possible.

The evaluation was initially designed to qualify the fidelity and implementation of the GPN program, identify any breach in the fidelity and suggest course correction, identify the frequency of participation and qualify target populations’ level of engagement, understand the perspective and experiences of participating girls and community members, and make program recommendations. The generalizability and transferability of the results may be limited when considering applications to other programs. Finally, WAGGGS groups girls into age categories that fall outside of the WHO's adolescent definition (17); thus, some results of this evaluation, particularly those obtained from WAGGGS’ youngest groups (i.e., 6–10 y), may not be generalizable to lessons about working with older adolescents or even adolescents in general.

### Conclusions

The results of this research may support and elaborate on several documented and accepted good practices for working with adolescents to improve nutrition through programming. The involvement of adolescents in the design, implementation, and evaluation of nutrition programming that targets them is essential for meaningful uptake. Sufficient time and respect must be given to the co-design process, including clearly defining adolescents’ roles, ensuring transparency and clear communication, and managing adolescents’ expectations. Ensuring adequate exposure and suitable timing for adolescent nutrition programming from adequately trained staff were identified as good practices from the evaluation. Program curriculum and activities must be appropriately tailored to adolescent age and stage, target adolescents and their gatekeepers and duty-bearers, and address the underlying issues of poverty, gender inequality, and structural norms, which negatively impact adolescents’ agency and nutrition. The results may also offer emerging good practices, such as increasing adolescent self-confidence and skills and engaging adolescents in influencing others on nutrition. More research is needed to tailor nutrition programs to support adolescents’ unique needs, including how best to ensure adolescent participation in all stages of nutrition programming.

## Notes

Nutrition International funded the program and the evaluation, delivered by Universalia Management Group, through the N-LIFT project, funded by Global Affairs Canada.

Author disclosures: The authors report no conflicts of interest. Nutrition International provided inputs in the design but did not participate in data collection or analysis.

Abbreviations used: CCT, co-creation team; FGD, focus group discussion; GG, Girl Guide; GPN, Girl Powered Nutrition; GS, Girl Scout; GSP, Girl Scouts in the Philippines; KII, key informant interview; LMIC, low- and middle-income country; MO, member organization; NI, Nutrition International; Universalia, Universalia Management Group; WAGGGS, World Association of Girl Guides and Girl Scouts.
